# Kawasaki disease shock syndrome with acute respiratory distress syndrome in a child: a case report and literature review

**DOI:** 10.1186/s12890-022-02007-w

**Published:** 2022-06-06

**Authors:** Jingwei Liu, Chunfeng Yang, Zhen Zhang, Yumei Li

**Affiliations:** Department of Pediatric Intensive Care Unit, The First Hospital of Jilin University, Changchun, 130021 China

**Keywords:** Kawasaki disease, Kawasaki disease shock syndrome, Acute respiratory distress syndrome

## Abstract

**Background:**

Kawasaki disease (KD) is an acute systemic vasculitis that may involve multiple organs. KD shock syndrome (KDSS) is a rare complication of KD. Pulmonary involvement is rare in KD; reports of patients with KD who develop KDSS and acute respiratory distress syndrome (ARDS) are extremely rare.

**Case presentation:**

A 2-year-old girl was brought to the emergency department with fever, cough and tachypnea. The patient was diagnosed with KDSS and ARDS. Extracorporeal membrane oxygenation (ECMO) and continuous blood purification were performed because of her critical condition. The patient eventually recovered completely. One year after discharge, there has been no coronary artery dilatation or pulmonary fibrosis.

**Conclusion:**

KDSS patients may develop ARDS due to fluid resuscitation and the release of inflammatory mediators. Early aggressive management and comprehensive treatment may improve prognosis.

## Background

Kawasaki disease (KD) is a multisystem vasculitis with mucosal and cutaneous manifestations. Pulmonary involvement, especially acute respiratory distress syndrome (ARDS), is rare in KD. KD shock syndrome (KDSS) is a rare manifestation of KD. KDSS is defined as the presence of systolic hypotension or clinical signs of poor perfusion accompanied by features of KD [1]. Reports of ARDS complicated by KDSS are extremely rare. Here, we summarize the diagnosis and treatment process of a patient with KDSS and ARDS.

## Case presentation

A 2-year-old girl presented to the emergency room with fever and cough that had lasted for 8 days and tachypnea for 4 days. She had developed a rash 5 days prior that gradually disappeared. On arrival, she was transferred to the pediatric intensive care unit for further treatment. On initial evaluation, the patient was lethargic. Body temperature was 38.5℃. Pulse was 156 beat/minute. Respiratory rate was 45/minute. Blood pressure was 85/40 mmHg.

She presented with strawberry tongue, bilateral conjunctival injection, unilateral cervical lymphadenopathy, and swollen feet. Auscultation of both lungs revealed rales. Laboratory examinations showed a C-reactive protein level of 219 mg/L, arterial partial pressure of oxygen of 57.6 mmHg, B-type natriuretic peptide of 2660 pg/mL, white blood cell count of 10.4 × 10^9^/L, hemoglobin level of 90 g/L, platelet count of 332 × 10^9^/L, creatine kinase isoenzyme 44.5 U/L, and albumin 21.7 g/L. Transaminase levels were normal, serum interleukin-4 was 9.95 pg/mL, interleukin-6 was 148 pg/mL, and interleukin-10 was 7.38 pg/mL. Sputum, blood, and urine culture turned out negative. The detections for viruses were negative, including SARS-CoV2 influenza virus, parainfluenza virus, respiratory syncytial virus and adenovirus. The admission electrocardiogram indicated sinus tachycardia. The admission echocardiography findings showed normal ejection fraction and diastolic function. The patient was diagnosed with severe pneumonia, respiratory failure, typical KD, and KDSS. Invasive mechanical ventilation, vasoactive drugs, intravenous immunoglobulin, and fluid resuscitation were initiated immediately; however, the patient’s condition worsened and progressed to ARDS.

**Fig. 1:**
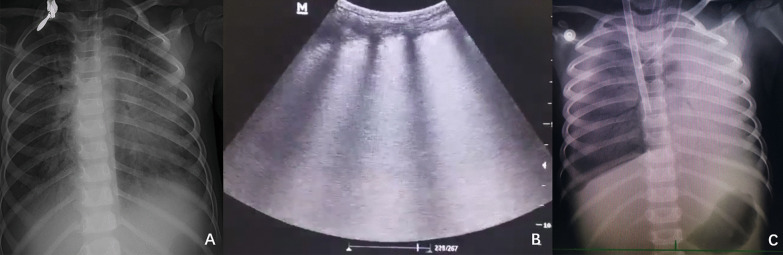
**A** The chest X-ray on the day before applying ECMO. **B** The lung ultrasound on the first day of applying ECMO. **C** The chest X-ray on the first day of applying ECMO

**Fig. 2:**
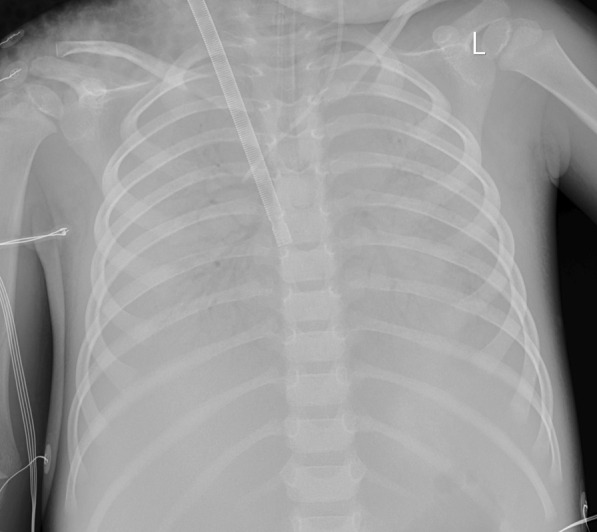
Chest X-ray during extracorporeal membrane oxygenation treatment diffuse density enhancement shadow in both lungs

The patient received high level of positive end-expiratory pressure (PEEP), prone positioning, recruitment maneuvers, and mechanical ventilation with strategies that limit tidal volumes and inspiratory pressures due to ARDS. At the beginning, the PEEP was 5 cm H_2_O. Due to severe ARDS, the PEEP was gradually increased. The maximal PEEP we provided for the patient was 15 cm H_2_O.We initiated continuous blood purification considering the severe inflammatory reaction of the patient. However, there was no significant improvement. Oxygenation index fluctuated between 25 and 36. The discontinuation of sedatives awakened the patient; she was then unconscious with less activity. Invasive mechanical ventilation was gradually ineffective. On day 4, veno-venous extracorporeal membrane oxygenation (ECMO) was initiated to provide oxygenation support. The chest X-ray and lung ultrasound were showed in Fig. 1. Arterial blood gas analysis and inhaled oxygen concentration before ECMO were showed in Table 1. During the preparation of ECMO, the oxygen supply is extremely poor. There was no heart sound during auscultation, the value of percutaneous blood oxygen could not be measured, the blood pressure decreased to 60/36mmhg, and the heart rate decreased to 60 beats per minute. Cardiopulmonary resuscitation and epinephrine were administered immediately. When the child was placed on ECMO, the blood pressure was 104/55 mmHg. On the second day after ECMO therapy, a chest X-ray showed a scattered patchy vague enhancement shadow (Fig. 2). There was a rapid clinical improvement over the next few days. The levels of cytokines (interleukin-4, interleukin-6, interleukin-10) and C-reactive protein decreased significantly.

**Table 1:** Arterial blood gas analysis and inhaled oxygen concentration before ECMO

Time	pH	PCO_2_	PO_2_	FiO_2_	P/F
D1	7.34	52.1	57.6	60	96
D2	7.39	47.2	98.9	50	197.8
D3	7.25	53.5	134.6	70	192.3
D4 (07:00)	7.29	52.3	93.5	70	133.6
D4 (15:00)	7.22	59.2	49.2	90	54.7

**Fig. 3:**
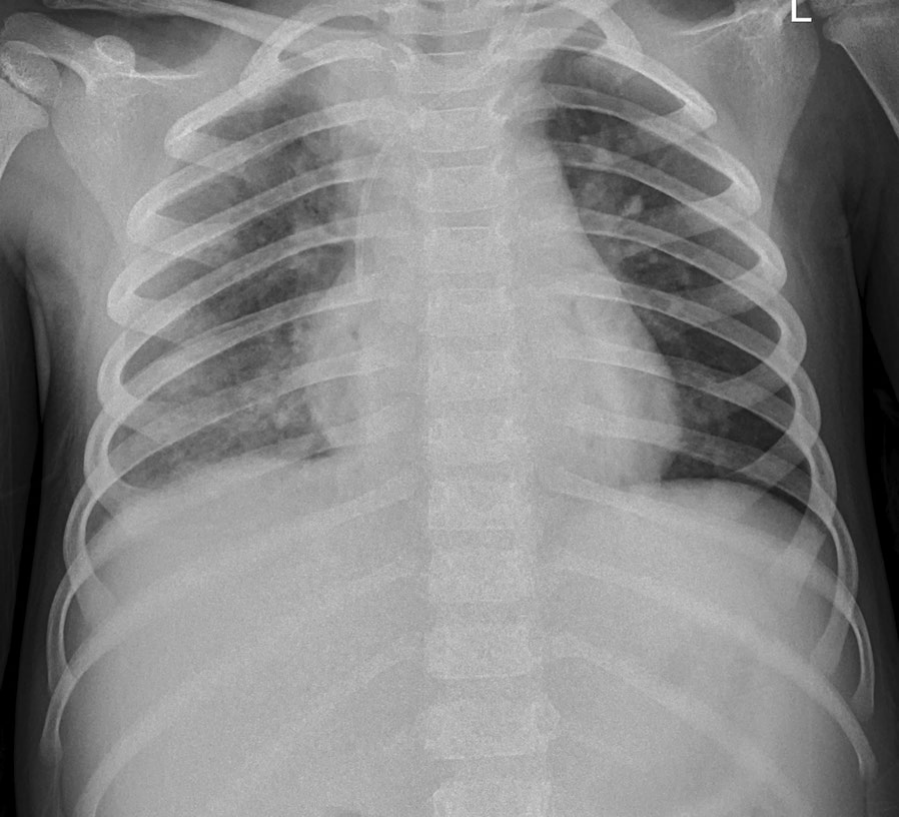
Chest X-ray after stopping ventilator assisted ventilation showed the inflammatory changes of both lungs were significantly improved

On day 8, continuous blood purification was stopped, and the patient’s respiratory failure gradually resolved. Some laboratory parameters during hospitalization were showed in Table 2. Procalcitonin dramatically increased on day 5 due to cardiopulmonary resuscitation. The platelets decreased dramatically due to ECMO and blood purification. The child had fever, so we monitor procalcitonin. The repeated cultures after day 5 were negative. The ECMO parameters gradually decreased. She was successfully weaned from ECMO and the ventilator on the 12th and 14th day of admission, respectively. Chest radiography after ventilator-assisted ventilation showed that the inflammatory changes in both lungs had significantly improved (Fig. 3). After the ventilator was stopped, the patient had clear consciousness and could communicate; her upper limb muscle strength was grade 3, and that of her lower limbs was grade 2. Brain MRI showed abnormal signals in the right temporal lobe and bilateral cerebellar hemisphere, a slightly widened and deepened sulcus and cistern, and a narrowed gyrus. Hyperbaric oxygen therapy and rehabilitation were initiated because of neurological damage. Eventually, the patient had a complete recovery from neurological symptoms. Echocardiography did not reveal any coronary artery dilatation, and the patient’s cardiac function was normal. On day 29, the patient was discharged with low-dose aspirin therapy. A year after discharge, the patient had not developed coronary artery dilatation or pulmonary fibrosis.

**Table 2:** Some laboratory parameters during hospitalization

Time	CRP (ng/L)	ALB (g/L)	PCT (ng/ml)	WBC (10^9^/L)	PLT (10^9^/L)	IL-4 (pg/ml)	IL-10 (pg/ml)
D1	219.8	–	–	12.75	383	–	–
D2	187.63	21.7	0.63	10.44	332	9.95	148.93
D3	190.35	24.8	1.14	21.95	217	–	
D4	205.59	–	1.1	19.35	122		
D5^a^	86.52	34.9	55.43	24.8	20		
D9	27.64	37.3	1.96	19.95	164	1.27	106.4
D14	7.42	42	0.19	9.77	251	–	
D20	8.15	–	0.08	4.56	673		
D23	33.35	–	0.32	5.34	432		
D29	9.15	49.9	–	7.22	407		

*CRP* C reactive protein, *ALB* albumin, *PCT* procalcitonin, *WBC* white blood cell, *PLT* platelet, *IL-4* interleukin-4, *IL-10* interleukin-10

^a^D5 was the second day of ECMO

– The laboratory parameter was not performed on that day

## Discussion and conclusion

Here, we describe a typical child with KD who developed KDSS and ARDS. Multisystem inflammatory syndrome in children (MIS-C), which emerged in the spring of 2020, is associated with SARS-CoV-2 [2]. Although the clinical presentations of our patient were similar to those of MIS-C, MIS-C was not considered due to the negative SARS-CoV-2 infection. KDSS refers to KD patients who show peripheral blood circulation perfusion disorder or present more than a 20% decrease in systolic blood pressure compared to healthy individuals of the same age [3]. KDSS is a rare complication of KD but can lead to poor outcomes. One study reported that 5% of patients with KD met the definition of KDSS [4]. The exact causes of KDSS are unknown and may include myocardial dysfunction, vasculitis with persistent capillary leakage, and cytokine dysregulation [1]. Patients with KDSS may have an uneven clinical course and may be misdiagnosed when first examined [5]. Early identification of KDSS is important for initiating appropriate and aggressive treatment, which reduces the risk of sequelae [6]. Patients with KDSS are prone to developing worse cardiac outcomes, gastrointestinal manifestations, incomplete presentation, and intravenous immunoglobulin resistance [4]. The clinical manifestations of KDSS include lymphadenectasis, hypoalbuminemia, anemia, hyponatremia, hepatic insufficiency, and electrocardiogram abnormalities [3]. Our patient presented with the typical clinical manifestations of KD, hypotension, anemia, and low protein levels; therefore, she was diagnosed with KDSS upon admission.

KD with pulmonary involvement is an uncommon condition. The lung manifestations in patients with KD include pneumonia [7], atelectasis, pleural effusion [8], pulmonary artery aneurysms [9], and pulmonary nodules [10]. The initial lung manifestation in our patient was pneumonia; however, she soon developed ARDS and required ECMO treatment. ARDS is a non-cardiac pulmonary edema characterized by rapidly progressive dyspnea, tachypnea, and hypoxemia [11].

The possible causes for KDSS developing ARDS in our patient may include fluid resuscitation and release of inflammatory mediators. In our case report, the patient with KDSS underwent fluid resuscitation, which may have aggravated ARDS. Early fluid loading transitorily can improve hemodynamics and oxygenation but worsen lung aeration [12]. ARDS occurs when pulmonary or extrapulmonary injury leads to the release of inflammatory mediators that promote inflammatory cell accumulation in the alveoli and microcirculation of the lungs [11]. Inflammatory cells damage the epithelium and result in pulmonary edema, pulmonary hyaline formation, lung compliance, and decreased gas exchange [11]. Patients with KDSS tend to produce more cytokines, which may play a role in developing systemic capillary leak syndrome [13]. In our patient, cytokines and C-reactive protein levels increased significantly; the release of inflammatory mediators may have played a very important role in developing ARDS. A study showed that vascular leakage might be a feature of KD [14]. We speculated that vascular leakage might be the cause of ARDS in children with KD.

Intravenous immunoglobulin and acetylsalicylic acid are the main treatments for KD [15]. Although our patient received these treatments, her condition was extremely critical, and treatment was very difficult. In addition to high level of PEEP, prone positioning, recruitment maneuvers, and mechanical ventilation with strategies that limit tidal volumes according to the ARDS guidelines [16], ECMO and continuous blood purification were used in our patient because of severe ARDS. Before ECMO treatment, the child was unconscious due to impaired oxygenation. When lung-protective strategies lead to insufficient gas exchange, children with severe ARDS should be considered for ECMO [17]. After performing ECMO, there was a significant improvement in the patient’s clinical condition. Although the patient was successfully weaned off ECMO and ventilator-assisted ventilation, she developed muscle weakness. Risk factors for muscle weakness in the ICU include immobility, multiorgan system failure, hyperglycemia, glucocorticoids, sepsis, persistent systemic inflammation, and neuromuscular blocking agents [18]. For patients with shock-induced ARDS, treatment with continuous blood purification in addition to standard therapies can improve lung function, hemodynamic stability, and endothelial function and reduce the length of mechanical ventilation [19]. After continuous blood purification treatment, our patient’s cytokine and C-reactive protein levels decreased significantly. A study showed that 50% of patients with KDSS require corticosteroid therapy [3]; however, the use of corticosteroids in children with KD has been controversial [15]. Corticosteroids were not administered for our patient. To the best of our knowledge, there is only one report of KD complicated by ARDS. However, that case report showed that a patient with KD complicated by ARDS maintained adequate oxygenation by maximum ventilator settings rather than ECMO [20]. Treatment strategies may vary depending on the disease severity. In the extremely critical situation of our patient, it was beneficial for the lungs to rest through ECMO and low ventilator parameters. We did our best to save this child’s life and also reduce ventilator-related and hyperoxia-related lung injury.

In conclusion, our case report describes the treatment and clinical manifestations of ARDS secondary to KDSS. KDSS patients may develop ARDS due to fluid resuscitation and the release of inflammatory mediators. The early identification of KDSS is very important. Early aggressive management and comprehensive treatment may improve the prognosis of ARDS induced by KDSS.

## Data Availability

The datasets used in the current study are available from the corresponding author upon reasonable request.

## Abbreviations

KD: Kawasaki disease
KDSS: Kawasaki disease shock syndrome
ARDS: Acute respiratory distress syndrome
ECMO: Extracorporeal membrane oxygenation
PEEP: Positive end-expiratory pressure
MIS-C: Multisystem inflammatory syndrome in children
